# Blood Tests for Alzheimer’s Disease: Increasing Efforts to Expand and Diversify Research Participation Is Critical for Widespread Validation and Acceptance

**DOI:** 10.3233/JAD-215730

**Published:** 2022-11-22

**Authors:** Thomas K. Karikari

**Affiliations:** a Department of Psychiatry and Neurochemistry, Institute of Neuroscience and Physiology, The Sahlgrenska Academy, University of Gothenburg, Gothenburg, Sweden; b Department of Psychiatry, University of Pittsburgh, Pittsburgh, PA, USA

**Keywords:** Alzheimer’s disease, amyloid-β, blood biomarker, dementia, diagnostics, low- and middle-income countries, minoritized populations, neurofilament light, phosphorylated tau

## Abstract

The recent academic and commercial development, and regulatory approvals, of blood-based Alzheimer’s disease (AD) biomarkers are breakthrough developments of immense potential. However, clinical validation studies and therapeutic trial applications are limited almost exclusively to non-Hispanic White cohorts often including highly-educated, high-earning participants. This commentary argues that the true benefits of blood tests for AD will be realized by active inclusion of diverse groups including minoritized populations, people of socioeconomic status different from those included in existing cohorts, and residents of low- and middle-income countries. The article discusses key factors that are critical for a successful implementation of diversity programs.

One of the most significant advancements in dementia research, diagnosis, and care in recent years is the development of blood-based biomarkers for Alzheimer’s disease (AD) [1–3]. There are now available high-performing blood tests for amyloid-β (Aβ_42_/Aβ_40_), phosphorylated tau (p-tau), and neurodegeneration (neurofilament light, NfL) that together show potential to translate the amyloid (A)/tau (T)/neurodegeneration (N) classification system [4] to blood [5]. These blood-based AT(N) biomarkers have demonstrated high accuracy and robustness to detect pathophysiological evidence of AD, showing strong agreements with neuropathological diagnosis and cognitive decline, as well as with the cerebrospinal fluid (CSF) and neuroimaging biomarkers that are currently used in specialized clinics [6–12]. These properties make blood biomarkers highly attractive for clinical diagnostic use, longitudinal monitoring, population screening, and therapeutic trials [3, 13, 14]. Given these exciting breakthroughs, it is unsurprising that the adaptation of research-grade blood tests into commercial products is moving rapidly, with several pharmaceutical and biotechnology companies actively involved [2, 3]. For instance, C2N Diagnostic’s plasma Aβ test has received Clinical Laboratory Improvement Amendments approval for *in vitro* diagnostic use while Quanterix’s Single molecule array (Simoa) p-tau181 assay has received an United States Food and Drugs Administration (FDA) Breakthrough Device designation [15].

The field is justifiably excited about the impact that blood tests will have— widespread access to diagnosis based on biological definition of the disease. Presently, CSF and neuroimaging biomarker assessments are limited mostly to Europe, North America, and Australia, cutting off most of the world accounting for > 80% of the global population [16, 17]. Even in these three continents, access to biomarker-supported dementia care is not universal; access to biomarker testing is limited by factors such as the availability of specialized imaging facilities or clinicians trained to perform lumbar punctures, willingness to undergo minimally invasive sampling or radiation exposure, and the financial capacity to afford the cost [3]. Switching to blood, the most ubiquitous biofluid in clinical chemistry, has the capacity to address these limitations. In theory, blood sampling can be performed anywhere (e.g., at the hospital, at home, or in the community). Unlike neuroimaging, retrospective blood analysis is possible provided samples are processed and stored properly. Blood testing is also simpler, more cost-effective and more acceptable to patients and study volunteers [13]. Moreover, disease changes tend to reflect earlier in blood (measuring soluble pathophysiological agents) than existing neuroimaging techniques (targeting protein/peptide aggregates that take several years to accumulate), allowing for detection of subtle changes very early in the disease process before symptoms develop [6, 14, 18, 19]. Another advantage of blood-based diagnostics is the use of samples processed using standard protocols, simplifying integration into existing clinical chemistry procedures at any hospital or research facility.

The aforementioned prospects of blood tests present a real and timely opportunity to broaden access to biomarker-supported AD diagnosis and management to yet-to-reach populations and environments. Leading experts rightly refer to this as “democratizing Alzheimer’s diagnostics” [20]. However, so far, clinical validation of blood biomarkers has been almost exclusively limited to research cohorts and therapeutic trials recruiting individuals who identify as non-Hispanic White [21, 22]. A recent review reported that only two [23, 24] of dozens of publications on blood p-tau to date included cohorts with diverse participants [3].

Predictably, it is the well-resourced medical centers (those with existing CSF and/or neuroimaging capacity) that have included or have started making arrangements to integrate blood testing into their healthcare systems. For example, blood NfL testing is available in major hospitals in Sweden and the Netherlands whereas blood Aβ and Aβ positron emission tomography (PET) assessments are accessible in some referral hospitals in the United States and Europe [25]. However, major centers expanding their options to biomedical care should not be assumed to necessarily address “widespread application.” This is because lack of financial affordability is a key limiting factor preventing many low-income households from accessing state-of-the-art healthcare, at least in the United States and other countries without universal healthcare.

This commentary article argues that for a fuller realization of the potentials of blood biomarkers, the field needs to look beyond where current access to biomarker-supported diagnosis is available and who can afford to pay for this service. This is what will help achieve the anticipated vision of widespread access.

Presently, what we know about AD and other dementias is principally from extensive genotyping and phenotyping of limited populations in the three most-studied continents— Europe, North America, and Australia [21, 22]. Apart from the majority of research cohort volunteers identifying as non-Hispanic Whites, these individuals also tend to be highly educated, live in neighborhoods with good social amenities, and have high-paying professional jobs that can provide the needed cushion for a healthy lifestyle [26, 27]. While blood tests will undoubtedly simplify and streamline the diagnostic procedures in these established settings and populations, more impactful potential may rest in expanding access to and actively reaching out to include populations currently without access or lack the means to afford such services. Participation of non-Hispanic Whites of other demographics and socioeconomic status (e.g., fewer years of education, less-paying and less-secure jobs, and live in less affluent or poorly resourced neighborhoods) in AD research is less frequent, presumably due to barriers identified in previous research [28, 29]. Including more people of these demographic features that differ from those of people often recruited into existing research cohorts would be a critical step to ensure that blood biomarker results reflect the larger population.

Another community of interest is minoritized populations who have historically been poorly studied and/or have complex histories around medical experimentation on humans that might contribute to their less likelihood to enroll in research studies involving invasive sampling [30, 31]. In the United States, minoritized populations include, but not limited to, Native Americans, Asian Americans, African Americans, Hispanic Americans, and Caribbean Americans, to name a few. In Europe, the concept of population diversity across ethno-racial perspective is less well-defined except in the United Kingdom where these populations are jointly referred to with terms such as Black, Asian and minority ethnic (BAME) although the Commission on Race and Ethnic Disparities has recommended against their use [32]. While it has long been assumed that the concentrations and clinical performances of biomarkers will be the same for people from all backgrounds, recent investigations have started to point out that what we thought of as universal knowledge may not be simply generalizable to other populations. For example, differences in the intensity of brain Aβ aggregates at the same disease stage have been reported between specific non-Hispanic White and African American populations [33] to suggest that there may be upstream factors modulating differential Aβ-PET uptake. Moreover, abnormality thresholds of the core CSF AD biomarkers Aβ_42_/Aβ_40_, p-tau and total-tau defined primarily in non-Hispanic White cohorts do not appear to be generalizable to people from other racial and ethnic backgrounds [22, 34, 35]. These biomarker disparities seem to translate to blood: a recent study of pairs of non-Hispanic White and African American participants matched for sex, age, *APOE* ɛ4 genotype, and cognition replicated these CSF and Aβ-PET findings [36]. Schindler et al. [36] further showed that the accuracy of plasma p-tau to predict abnormal brain Aβ tends to differ according to race/ethnicity. Another study suggests that blood-based neurodegeneration biomarker profiles, assessed using plasma NfL, differ in Hispanic-White versus non-Hispanic-White Americans [37]. These results, paralleling observations from other areas of medicine [38], suggest that biomarker cut-offs may not be readily transferable. The findings further suggest that studying diverse populations is a unique opportunity to better understand and characterize the complexities of the disease, and its potential interactions with environmental and lifestyle factors that oftentimes vary on racial and ethnic lines [39]. A potential way to approach this is to carefully study different and representative racial and ethnic groups throughout the world to be able to determine if these biomarkers perform the same way in all individuals and if abnormality thresholds are transferrable.

Dementia affects people globally [40], yet statistics from low- and middle-income countries (LMICs) are incomplete and are usually extrapolated from other countries’ data [41]. Importantly, dementia and AD incidence estimates are mostly without biomarker confirmation. Hence, the pathophysiological profiles are expected to differ to an extent from the clinical presentation (because clinical diagnosis disagrees with autopsy-verified AD by 30% [42]). As biomarker-supported dementia diagnosis and care is lacking in LMICs [41], it is presently unknown if blood biomarker performances are generalizable to these settings. Given differences in the social and health exposures across the life course (e.g., social determinants of health, medical comorbidities, cardiovascular disease, genetic risk variants, stress, diet, physical activity) between some LMICs and high-income countries [43] that are shown to modulate/associate with AD biomarker changes [39], it is conceivable that these associations could vary by environment.

Dementia researchers, clinicians, public health experts, and policy makers worldwide should actively and consciously devise programs to expand access to blood tests for AD for both research and clinical purposes. Such efforts will need to identify and address issues that will be critical for a successful implementation. The following clinical, analytical, and ethical factors will be important:

a. *Let*’*s face it, health disparities exist.* It may be easy to assume that everything is fine, however accumulating data from multiple fields and perspectives continue to show that the reality concerning health disparities is the opposite [26, 44]. A sustainable way forward is to first admit that disparities in healthcare exist across racial, ethnic, and socioeconomic divides— for example in healthcare seeking behaviors, access to healthcare, affordability, and the social aspects of health. The next step will be to try to understand why and how such disparities arise, to provide valuable information as to how to bridge these gaps. At least, countries like the United States have started to grapple with the situation and are institutionalizing efforts to better understand and address them. Although health disparities in places like Europe may not be as aggravated as in the United States [45], starting to address such inequalities now instead of later would be a step in the right direction.
b. *Outreach initiatives to explain benefits and allay fears.* Some populations and communities, including minoritized groups, may require carefully designed outreach engagement programs to dialogue on the purpose, benefits, and risks of volunteering for medical research involving biospecimen donation.
c. *Mutually beneficial collaborative research*. Comprehensive understanding of the (patho)physiological, social, environmental, and epigenetic factors that modulate biomarker dynamics in health and disease will require in-depth appreciation of the way of life of the people being studied. For this to happen, efforts must be made to avoid “helicopter research”— the concept where scientists from high income countries visit LMICs to collect samples and leave without meaningful partnerships with local scientists [46].
d. *Engaging and training primary care physicians to integrate biomarker assessments into their clinical algorithms*. As highlighted in the Alzheimer’s Association’s 2020 facts and figures [47], healthcare in many communities suffer from acute shortages of specialists in dementia care trained and experienced in detecting clinical signs and symptoms of cognitive decline. These deficiencies may be associated with the paucity of epidemiological statistics from these settings [41]. An important advantage of blood testing is that the results may be interpretable by primary care physicians and other non-dementia specialists for the purpose of identifying patients who may need specialist attention for suspected cognitive decline. This way, straightforward cases (e.g., biomarker-positive AD dementia, biomarker-negative non-AD dementia) may be preliminarily diagnosed by corroborating blood biomarker results with clinical presentation while patients presenting with less clear profiles are referred to specialist care [3]. Such a streamlined approach might reduce the pressure on the few secondary and tertiary care facilities available in these settings. However, there is a reasonable level of risk associated with this proposal because relying heavily on blood biomarker results without comprehensive dementia evaluation might also lead to a good number of false positive cases. However, one can argue that such false positivity would be rectified once a complete dementia assessment is done by specialists at referral centers.
e. *A positive blood biomarker profile does not necessarily indicate AD.* One of the principal applications of blood biomarkers is the prognosis of the risk of developing AD. Since plasma p-tau, Aβ_42_/Aβ_40_, and glial fibrillary acid protein (GFAP) results associate with Aβ PET [6, 8, 10, 48, 49], abnormal concentrations of these biomarkers are likely to be interpreted as high risk of developing AD in the future. However, a significant proportion of cognitively individuals with Aβ-PET positivity do not develop cognitive impairm22-04-2022ent several years later. By extension, many people with abnormal plasma biomarker profiles are expected not to develop future clinical signs of AD dementia. It is therefore essential to factor such conservative estimates in prediction algorithms based on plasma biomarker results, and to consider family history and genetic predispositions (e.g., *APOE* ɛ4 status) in risk prediction.
f. *How useful is AD biomarker screening without access to drug interventions?* A justified criticism of blood-based screening for AD is how useful it is in practical terms when availability of, and access to, drug interventions is acutely limited. The FDA-approved Aduhelm® is cost-prohibitive to low-income households even in the United States, and thus the majority of residents in LMICs where no such drug is even approved for clinical use. Furthermore, it remains unknown how effective the drug is in people from ethnically and racially diverse backgrounds due to their poor representation in the clinical trials leading to its approval [50]. Moreover, since dementia is not seen as a disease and often goes undetected in many communities, it may be challenging to convince such populations that clinical symptoms are treatable [51].
g. *Efforts to offset costs of biomarker testing*. As screening for some common cancers and non-communicable diseases is offered free of charge or subsidized to individuals in the risk age group, it can be envisaged that in the near future blood screening tests for AD will be offered to elderly adults. Successful implementation of these projects will require that costs are subsidized especially for low-income populations.
h. *Blood sample handling in remote/rural settings*. Since settlements tend to be more dispersed in rural communities, distance to a health facility or centralized laboratory may vary significantly, and in-person hospital attendance may not be feasible in all circumstances. This point may even apply to urban areas during unusual circumstances like pandemics. For field collection of blood, a critical point to consider is the pre-analytical handling. Transporting samples from the field to the laboratory can prolong the usual time from blood collection to further processing. It also deviates from other standard conditions of handling (e.g., temperature, storage, shipping). These divergences may not affect all samples in the same way— samples delivered to the laboratory in an hour versus five hours may be affected differently. Furthermore, in environments lacking reliable electricity access, long-term storage in ultra-cold conditions will be challenging. For these reasons, standardized methods for blood collection and handling specifically adapted to rural/sparse settlement settings will be needed. Emerging methods that circumvent centrifugation and freezing (both requiring electricity) may be further explored [52].
i. *Ethics of disclosing biomarker results to patients and caregivers*. Dementia is still viewed with stigmatization even in some high-income countries [53]. Therefore, disclosing biomarker positivity results or giving one a diagnosis of AD is likely to present with its own peculiar challenges and ethical considerations. The ethics are likely to vary between populations and according to disease status. Concerning populations, it is anticipated that the number and variety of concerns raised may be more in minoritized communities with less previous research engagements. When considering disease stage, those diagnosed with symptomatic AD may be primarily interested in exploring why and how they developed the disease and the available remedial interventions while elderly adults with normal cognition may focus on understanding their risk of future cognitive impairment. It would thus be wise to perform dedicated research on this subject to understand how best to disclose and manage dementia diagnoses.
j. *Participation in clinical trials for AD.* Awareness is increasingly being created that clinical trial participants should be diversified. Anticipated challenges to achieving this goal include difficulties to recruit volunteers willing to undergo periodic CSF or PET assessments. This is one means where blood-based biomarkers will find a unique application. In addition to reducing financial cost for pharmaceutical companies, blood biomarkers may improve participation from some groups that have traditionally been reluctant to undergo more invasive forms of biomarker testing. Recent studies have shown that pre-screening with blood biomarkers can help to identify a target group of individuals who fit the desired profile (amyloid positivity) for more comprehensive clinical examination [3, 13]. Indeed, some companies have already started to put this into practice. For example, trials of the anti-amyloid drugs aducanumab and donanemab reported 13–16% and 24% group-level reductions in plasma p-tau181 and p-tau217 respectively in agreement with observed amyloid clearances [54, 55]. Having shown that blood-based p-tau could be a surrogate marker for brain amyloid clearance in trials that target Aβ, it may be a realistic future expectation to see therapeutic trials that depend on blood biomarkers for inclusion and monitoring. This would be a welcome development to increase participation of populations for whom blood-based diagnostics are the only logistically feasible means to be evaluated for AD. However, for this to happen, there is the crucial need to validate blood biomarkers across multiple populations, as discussed above.
k. *How can blood biomarkers be validated in populations without access to gold-standard methods?* Firstly, neuropathological confirmation of AD has been shown across diverse racial and ethnic groupings, meaning that primarily AD is pathologically characterized by plaques and tangles [56, 57]. Secondly, one does not need access to PET or CSF biomarkers to validate blood biomarkers in diverse settings especially since these established biomarkers have already been validated against neuropathology in the mostly White cohorts and a few diverse cohorts. The issue of primary concern, however, is to answer the question if blood biomarker concentrations and clinical performances are the same when comparing people with shared demographic features (e.g., age, sex, cognition, *APOE* ɛ4 genotype, or comorbidities). The importance of this issue is partly highlighted by differences in concomitant pathologies, both neurodegenerative and other diseases, observed across racial and ethnic lines [56, 57].

In conclusion, the recent successes in blood biomarkers for AD present the field with opportunities to truly expand access to diagnosis and research, and to better understand the disease from multiple perspectives. The success of these expansion and diversification efforts may be contingent on the ability to translate these prospects to environments and populations that are often outside the radar of biomarker-supported medicine. To achieve this requires the development and implementation of specific programs adapted to the needs and individualities of the target communities and populations, in partnership with local experts.
